# Peripheral Immune-Mediated Neuropathy Before and During the COVID-19 Pandemic: A Retrospective Cross-Sectional Study in a Referral Center

**DOI:** 10.7759/cureus.74131

**Published:** 2024-11-21

**Authors:** Michael Angelo O Su, Maria Leila M Doquenia, Michelle J Tanglao, Raymond Rosales

**Affiliations:** 1 Department of Internal Medicine, Metropolitan Medical Center, Manila, PHL; 2 Center for Neurodiagnostic and Therapeutic Services, Metropolitan Medical Center, Manila, PHL; 3 Research Center for the Health Sciences, University of Santo Tomas, Manila, PHL; 4 Institute for Neurosciences, Saint Luke's Medical Center, Quezon City, PHL

**Keywords:** chronic inflammatory demyelinating polyradiculopathy, cidp, dm neuropathy, gbs, guillain-barre syndrome, peripheral immune-mediated neuropathy

## Abstract

Background: There are currently seven coronaviruses that can infect humans and the latest addition to these viruses is the severe acute respiratory syndrome coronavirus-2 (SARS-CoV-2). Infection by SARS-CoV-2 is known commonly as coronavirus disease 2019 (COVID-19). Aside from common manifestations of cough and fever, neurologic symptoms such as headache, disturbed consciousness, paresthesia, and seizures have also been seen. By identifying the incidence of peripheral immune-mediated neuropathies (PIMN), early prevention can be made.

Methods: This cross-sectional retrospective analytic study reviewed the case records of patients examined at the Center for Neurodiagnostic and Therapeutic Services (CNS) of Metropolitan Medical Center from January 2018 to December 2021.

Results: The period incidence of Guillain-Barré Syndrome (GBS) for the years 2018-2019 and 2020-2021 were 9.21% and 24.44%, respectively. The obtained p-value was 0.0226, which was lower than the set p-value of 0.05. Therefore, the period incidence of GBS for 2020-2021 was significantly higher when compared to that of 2018-2019.

Conclusion: There has been an increase in cases of GBS during the COVID-19 pandemic.

## Introduction

Coronaviruses are large, enveloped, positive-sense RNA viruses. There are currently seven coronaviruses that can infect humans and the latest addition is the severe acute respiratory syndrome coronavirus-2 (SARS-CoV-2). This is a virus identified in Wu Han China in December 2019 leading to the coronavirus disease 2019 (COVID-19) pandemic.

Coronaviruses have been associated with neurologic manifestations. An example of this is SARS-CoV-1, identified in 2002-2003, which has been associated with at least three reported cases of axonal variant Guillain-Barré Syndrome (GBS) and five cases of ischemic stroke [1]. Another virus under the family coronavirus known as Middle East Respiratory Syndrome CoV (MERS-CoV), identified in 2012, has also been reported to cause at least three cases of GBS [1].

SARS-COV-2 shares a close sequence homology to SARS-CoV-1 using spike proteins on the viral surface reacting to angiotensin-converting enzyme (ACE) receptors on mammalian hosts. These receptors are found mostly in airway epithelia, kidneys, small intestine, vascular endothelial, neurons, astrocytes, and oligodendrocytes causing symptoms associated with each site. SARS-CoV-2 causes viral neuro-invasion plausibly by trans-synaptic spread or blood-brain barrier (BBB) spread [1].

In a review by Shabani, the demolition of the myelin sheath was reported as a neurological complication of severe COVID-19 [2]. There are reportedly two possible entry modes into the CNS: via the cribriform plate/olfactory bulb route or via the hematogenous route. In the cribriform plate and olfactory bulb routes, the virus spreads through the cranial nerves, specifically the olfactory nerve, trigeminal nerve, glossopharyngeal nerve, and vagus nerve, via retrograde axonal transport.

The most widely accepted pathway is the ACE2 receptor pathway. The SARS-CoV-2 via the hematogenous route spreads to the cerebral circulation wherein there is a slower blood flow in the capillaries. Due to the slow blood flow rate, damage to the capillary endothelium accelerates thus making the brain accessible. ACE2 receptors are abundant in human endothelial cells, neurons, respiratory epithelia, pulmonary parenchyma, small bowel cells, renal cells, and nerve cells. Once the SARS-CoV-2 virus enters the BBB, it will bind with the abundant ACE2 receptors in the nerves via attachment between the spike glycoprotein and the ACE2 receptors. And with the BBB destroyed, direct virus contact is possible [2].

The CNS can also be affected indirectly through cytokines. In the severe form of COVID-19 infection, it may lead to an abnormal or aggravated immunological host response, resulting in severe systemic damage. There are three stages in the immunopathological pathway of COVID-19 mortality. Stage I is the initiation phase wherein there are early signs of systemic inflammation with early induction of chemokines and decreased peripheral leukocyte counts. Stage 2 is the amplification stage wherein there is an increased production of the inflammatory cells. Stage 3 is the consummation phase where the neutrophil count further increases while there is worsening lymphopenia and continuous increase of inflammatory mediators and cytokine storm leading to widespread organ damage. With the cytokine storm, these target the CNS because the released cytokines can cross the BBB and in turn activate the toll-like receptors (TLR) leading to the release of several interleukins, interferons, and pro-inflammatory cytokines. Some of the cytokines produced lead to the death of the neurons and oligodendrocytes and subsequently demyelination [2].

A case report by Zanin et al. in 2020 described a 54-year-old woman with severe COVID-19 infection presenting with seizures on electroencephalogram (EEG); brain magnetic resonance imaging (MRI) revealed alterations of the periventricular white matter, hyperintense T2 weighted image (WI), with neither restricted diffusion nor contrast enhancement [3]. Similar lesions were also found at the bulbo-medullary junction and in both the cervical and dorsal spinal cord. Cerebrospinal fluid (CSF) analysis was normal. The researchers were able to rule out multiple sclerosis, viral encephalitis, and bacterial CNS infection. However, due to the fact that brain MRI showed new onset, multiple, non-enhancing demyelinating lesions, they speculated that this might be caused by the existing COVID-19 infection [3].

Another report of a demyelinating disease of the CNS was described by Zhuang and Miskin from Thomas Jefferson University Hospital [4]. They described the first case of neuromyelitis optica (NMO) triggered by SARS-COV-2 infection. NMO was diagnosed based on the presence of at least one of six core clinical characteristics and detection of NMO/aquaporin (AQO) 4 IgG. The patient met the criteria for NMO based on longitudinally extensive transverse myelitis and area postrema syndrome in the setting of positive NMO/Q1P-4 serum antibodies.

There are also reported cases of acute central and peripheral demyelinating diseases after the administration of the COVID-19 vaccine. A total of seven cases was reported by Khayat-Khoei et al. in their case series, where two patients were reported to have acute demyelinating disease after the first dose of mRNA COVID-19 vaccination, and five patients presented with symptoms after the second dose of the vaccination, most of which happened within 1-21 days post vaccination [5]. Symptoms included visual loss, dysmetria, gait instability, paresthesia, sphincter disturbances, and limb weakness. All patients returned to baseline after high-dose IV steroids.

A study by Al-Mazidi and Al-Dakhil stated that due to the increasing neurological manifestation of COVID-19 patients, many neurologists came up with a protocol for evaluating COVID-19 peripheral neuropathies [6]. Combining clinical assessment, history of illness, and validated objective clinical assessment including electrophysiological assessment was suggested. They studied two common electrophysiological methods were studied, nerve conduction studies (NCS) and electromyogram (EMG). Al-Mazidi and Al-Dakhil concluded that COVID-19 generates a demyelinating motor neuropathy and myopathy wherein clinicians are encouraged to refer patients presenting with neurologic symptoms to be assessed by electrophysiological methods, and to objectively determine the nature of the illness, the prognosis, and plan for rehabilitation of COVID-19 patients [6].

This study aims to identify the incidence of acute demyelinating disease, specifically GBS, during the COVID-19 pandemic. There were many reported cases of neurologic sequelae of COVID-19, specifically peripheral immune-mediated neuropathies (PIMN), exemplified by GBS. However, the COVID-19 response in the Philippines focussed on the respiratory symptoms, neglecting the possible neurologic manifestations and consequences until severe. By identifying the incidence of PIMN, early prevention strategies can be developed.

## Materials and methods

This cross-sectional retrospective analytic study reviewed the case records of patients examined at the Center for Neurodiagnostic and Therapeutic Services (CNTS) of Metropolitan Medical Center from January 2018 to December 2021. the study was approved by the Ethics Review Committee of the University of the East Ramon Magsaysay Memorial Medical Center, Incorporated Research Institute for Health Sciences (approval number: 1263/E/2022/073).

Inclusion and exclusion criteria

Inclusion criteria were: Patients newly diagnosed with PIMN, exemplified by GBS and its variants via electrodiagnosis from 2018 to 2021 in the CTNS of Metropolitan Medical Center. Exclusion criteria were diagnosed cases of GBS before 2018.

Data collection

Data collection was done in the CNTS of Metropolitan Medical Center. A list of newly diagnosed patients with PIMN was obtained via chart review, then tabulated and classified according to the year as (i) Pre-COVID-19 Group (2018-2019) and (ii) COVID-19 Group (2020-2021). Records of patients were retrieved and the following data was obtained: age, sex, year of diagnosis, and diagnosis of PIMN and other peripheral neuropathies. The incidence rate for two years was then calculated.

Data analysis

A comparison of the incidence rate of the pre-COVID-19 group with the COVID-19 group was done. The data were stored in an Excel file (Microsoft Corporation, Redmond, Washington, United States) and discarded after the completion of the study. Data analysis consisted of descriptive and inferential statistics. Descriptive statistics were used to describe the demographic and clinical characteristics of included cases. Qualitative and quantitative data were numerically expressed as frequencies, proportions, and means ± SD, respectively. The sex ratio for chronic inflammatory demyelinating polyradiculopathy (CIDP) and GBS were obtained. Furthermore, the incidence of CIDP, GBS, diabetes mellitus (DM) neuropathy, and facial neuropathy (Bell’s Palsy) for the pre-COVID-19 and COVID-18 groups were compared using the comparison of two proportion z-tests, with a 95% confidence interval (CI). A p-value of <0.05 was considered statistically significant.

## Results

A total of 121 patients were included in the study, of which 58.68% (n = 71) were aged ≥ 60 years compared to 41.32% (n = 50) aged below 60 years, with slightly more female participants (n = 62, 51.24%) than male (n = 59, 48.76%). As shown in Table 1, the most common disease pathology encountered with peripheral neuropathy was diabetes mellitus neuropathy (n = 73, 60.33%) followed by facial neuropathy/Bell’s Palsy (n = 24, 19.83%), GBS (n = 18, 14.88%), and CIDP (n = 6, 4.96%). 

**Table 1:** Demographics and clinical data of study population (N=121) CIDP: chronic inflammatory demyelinating polyradiculopathy; GBS: Guillain-Barré syndrome; DM: diabetes mellitus

Characteristics	Percentage (Frequency)
Age	
< 60 years old	41.32% (n = 50)
≥ 60 years old	58.68% (n = 71)
Sex	
Male	48.76% (n = 59)
Female	51.24% (n = 62)
CIDP	
Yes	4.96% (n = 6)
No	95.04% (n = 115)
GBS	
Yes	14.88% (n = 18)
No	85.12% (n = 103)
Facial Neuropathy (Bell’s Palsy)	
Yes	19.83% (n = 24)
No	80.17% (n = 97)
DM Neuropathy	
Yes	60.33% (n = 73)
No	39.67% (n = 48)

The incidence of CIDP as well as the incidence of GBS in the pre-COVID-19 and COVID-19 groups were obtained and categorized according to age and sex (Table 2). Even though there were more patients seen during the year 2018-2019 (Pre-COVID-19 group), it can be noted that the year 2020-2021 (COVID-19 group) exhibited higher percentages of PIMN in all aspects. Comparing the variables in both timelines, only those in the ≥ 60 years and male categories demonstrated a p-value of less than 0.05. 

**Table 2:** Incidence of PIMN (CIDP and GBS) with respect to age and sex in the study groups CIDP: chronic inflammatory demyelinating polyradiculopathy; GBS: Guillain-Barré syndrome; PIMN: peripheral immune-mediated neuropathies; DM: diabetes mellitus Percentage given is that of participants with the disease among the specific age/sex groups p-value <0.05 is significant

	Pre-COVID-19 group (2018 – 2019) (n=76), % (n)	COVID-19 group (2020 – 2021) (n=45), % (n)	z score	p-value
Age < 60 years	17.86% (5/28)	36.36% (8/22)	-1.4809	0.06944
Age ≥ 60 years	10.42% (5/48)	26.09% (6/23)	-1.7077	0.04363
Male	10.52% (4/38)	28.57% (6/21)	-1.7688	0.03836
Female	15.79% (6/38)	33.33% (8/24)	-1.6093	0.0537

Table 3 shows the incidence of CIDP, GBS, facial neuropathy, and DM neuropathy with respect to age, as detailed above. Only the CIDP category had a p-value of greater than 0.05, while the rest of the p-values were less than 0.05. Therefore, the incidence of GBS and facial neuropathy among those aged < 60 years old were significantly higher compared to those aged ≥ 60 years. Furthermore, the incidence of DM neuropathy among those aged ≥ 60 years was significantly higher compared to those aged < 60 years.

**Table 3:** Incidence of CIDP, GBS, facial neuropathy (Bell’s palsy), and DM neuropathy according to age in the study population (N=121) CIDP: chronic inflammatory demyelinating polyradiculopathy; GBS: Guillain-Barré syndrome; DM: diabetes mellitus p-value <0.05 is significant

Neuropathies	< 60 years (n = 50), % (n)	≥ 60 years (n = 71), % (n)	z score	p value
(+) CIDP	2% (n = 1)	7.04% (n = 5)	-1.2581	0.10383
(+) Guillain-Barré Syndrome	24% (n = 12)	8.45% (n = 6)	2.3668	0.00889
(+) Facial Neuropathy (Bell’s Palsy)	26% (n = 13)	1.41% (n = 1)	4.1643	p < 0.00001
(+) DM Neuropathy	28% (n = 14)	83.10% (n = 59)	-6.1005	p < 0.00001

Table 4 shows the incidence of CIDP, GBS, facial neuropathy, and DM neuropathy with respect to sex, as mentioned above. All the p-values obtained are higher than the set p-value of 0.05. Therefore, there was no significant difference in the Incidence of the aforementioned disease pathologies with respect to sex.

**Table 4:** Incidence of CIDP, GBS, facial neuropathy (Bell’s palsy), and DM neuropathy according to sex in the study population (N=121) CIDP: chronic inflammatory demyelinating polyradiculopathy; GBS: Guillain-Barré syndrome; DM: diabetes mellitus p-value <0.05 is significant

Neuropathies	Male (n = 59), % (n)	Female (n = 62), % (n)	z score	p-value
(+) CIDP	6.78% (n = 4)	3.22% (n = 2)	0.9001	0.18406
(+) GBS	10.17% (n = 6)	19.35% (n = 12)	-1.4192	0.0778
(+) Facial Neuropathy (Bell’s Palsy)	16.95% (n = 10)	22.58% (n = 14)	-0.7765	0.2177
(+) DM Neuropathy	66.10% (n = 39)	54.84% (n = 34)	1.2659	0.10204

Of the 121 included patients, six had CIDP, 18 had GBS, 73 had DM neuropathy, and 24 had facial neuropathy (Table 5). The frequency of the disease in relation to sex and timeline was tabulated. The CIDP and DM neuropathy subsets had sex ratios greater than 1 in both timeline groups (2018-2019 vs. 2020-2021), garnering sex ratios for the total population of 2 and 1.15, respectively. This showed a slight predisposition of male participants for the aforementioned diseases. The GBS and facial neuropathy subsets, on the other hand, had sex ratios of less than 1 in both timeline groups (2018-2019 vs. 2020-2021), garnering sex ratios for the total population of 0.5 and 0.71, respectively. Hence female participants had a slight preponderance for the aforementioned diseases.

**Table 5:** Sex ratio according to CIDP, GBS, facial neuropathy (Bell’s palsy), and DM neuropathy in the two groups CIDP: chronic inflammatory demyelinating polyradiculopathy; GBS: Guillain-Barré syndrome; DM: diabetes mellitus

Neuropathies	Pre-COVID-19 group (2018 – 2019)	COVID-19 group (2020 – 2021)	Total (2018-2021)
(+) CIDP (n = 6)	Male: 2	Male: 2	Male: 4
	Female: 1	Female: 1	Female: 2
	Sex Ratio: 2	Sex Ratio: 2	Sex Ratio: 2
(+) GBS (n = 18)	Male: 2	Male: 4	Male: 6
	Female: 5	Female: 7	Female: 12
	Sex Ratio: 0.4	Sex Ratio: 0.57	Sex Ratio: 0.5
(+) Facial Neuropathy (Bell’s Palsy) (n = 24)	Male: 5	Male: 5	Male: 10
	Female: 8	Female: 6	Female: 14
	Sex Ratio: 0.625	Sex Ratio: 0.83	Sex Ratio: 0.71
(+) DM Neuropathy (n = 73)	Male: 29	Male: 10	Male: 39
	Female: 24	Female: 10	Female: 34
	Sex Ratio: 1.21	Sex Ratio: 1	Sex Ratio: 1.15

The period incidence of CIDP for 2018-2019 and 2020-2021 were 3.95% and 6.67%, respectively (Table 6). The obtained p-value was 0.50286, which was higher than the set p-value of 0.05. There was therefore no significant change in the incidence of CIDP in the Pre-COVID-19 group (2018-2019) when compared to that of the COVID-19 group (2020-2021). The period incidence of GBS for 2018-2019 and 2020-2021 was 9.21% and 24.44%, respectively. The obtained p-value is 0.0226, which was lower than the set p-value of 0.05. Therefore, the period incidence of GBS for 2020-2021 was significantly higher than that for 2018-2019.

**Table 6:** Incidence of CIDP, GBS, facial neuropathy (Bell’s palsy), and DM neuropathy in the two groups CIDP: chronic inflammatory demyelinating polyradiculopathy; GBS: Guillain-Barré syndrome; DM: diabetes mellitus p-value <0.05 is significant

Neuropathies	Pre-COVID-19 group (2018 – 2019) (n = 76), % (n)	COVID-18 group (2020 – 2021) (n = 45), % (n)	z score	p-value
(+) CIDP	3.95% (n = 3)	6.67% (n = 3)	-0.6659	0.50286
(+) GBS	9.21% (n = 7)	24.44% (n = 11)	-2.2759	0.0226
(+) Facial Neuropathy (Bell’s Palsy)	17.11% (n = 13)	24.44% (n = 11)	-0.9785	0.32708
(+) DM Neuropathy	69.74% (n = 53)	44.44% (n = 20)	2.7486	0.00596

The period incidence of facial neuropathy for 2018-2019 and 2020-2021 were 17.11% and 24.44%, respectively (Table 6). The obtained p-value was 0.32708, which was higher than the set p-value of 0.05. This shows that here was no significant change in the incidence of facial neuropathy in the Pre-COVID-19 group (2018-2019) compared to that of the COVID-19 group (2020-2021). On the contrary, the period incidence of DM neuropathy for 2018-2019 and 2020-2021 were 69.74% and 44.44%, respectively. The obtained p-value is 0.00596, which was lower than the set p-value of 0.05. Therefore, the period incidence of DM neuropathy for 2020-2021 was significantly lower than that for 2018-2019.

## Discussion

The family of coronaviruses has been identified to have caused neurologic abnormalities [1]. With coronaviruses sharing a close homologous sequence in their proteins, this study aimed to identify the incidence of GBS during the initial two years of the new COVID-19 virus and compare this with earlier incidence. Hence, all patients diagnosed with PIMN in 2018-2021 were compiled and reviewed.

This study concluded that the incidence of PIMN in the age group of ≥ 60 years and male categories in 2020-2021 was significantly higher when compared to that in 2018-2019. Furthermore, it could also be concluded that the incidence of DM neuropathy among those aged ≥ 60 years was significantly higher compared to those aged < 60 years.

According to a review by Kuwabara, GBS occurs in all ages with peak incidence seen in late adolescence and young adulthood, and there is a slight male preponderance with a male-to-female ratio of 1.25:1 [7]. In the present study, in general, PIMN was more commonly seen in patients under 60 years of age, while DM neuropathy was more commonly seen in those aged above 60 years. As for sex preponderance, the study shows a similar result to that of Kuwabara [7], with GBS more commonly seen in male patients. An increase in the incidence of GBS was seen in the COVID-19 period (2020-2021). This coincides with the study of Valaparla et al., which stated that the incidence of GBS increased during the pandemic as compared to the pre-pandemic period for the same month [8].

However, other types of PIMN such as CIDP did not significantly increase. DM neuropathy was the most common peripheral neuropathy, especially among male patients aged above 60 years. It also showed a significant decrease during the COVID-19 study period which may be attributed to lesser consultation of patients due to the pandemic. Moreover, a study published by Ordriozola concludes that a proportion of patients with diabetes and severe COVID-19 may develop or show worsening peripheral neuropathy [9]. 

A study done by Pimentel et al. also stated a possible relationship between COVID-19 and GBS due to overlapping symptoms of generalized weakness, reflex reduction, facial paresis/paralysis, hypoesthesia, and paresthesia [10]. This coincides with the results of the current study where the mean age of the patients diagnosed with GBS was 61 years and most were males. This was also confirmed in the review by Taga and Lauria, which calculated the male-to-female ratio as 1.6 (57:35) and the mean age as 55.2 ± 17.3 years [11]. 

In a study by Trentinaglia et al., patients with COVID-19 underwent serological testing to diagnose different PIMN [12]. In their study, GBS was considered one of the peripheral neuropathy triggered by the COVID-19 infection. However, their study also showed that CIDP is as common as GBS among those infected with COVID-19.

The COVID-19 vaccine associated with GBS was not included in this study. However, several studies have shown that vaccination is linked to GBS. One study showed the rate of GBS from COVID-19 vaccination was 1.8-53.2 cases per one million doses with an average time from vaccination to symptoms of 11.2 days [13]. In another systematic review and meta-analysis, the mean interval from vaccination to GBS symptom development was 13.0 ± 6.9 days, ranging from one to 37 days, and most symptoms occured after the first dose of the vaccine [14]. However, any neurologic symptoms from COVID-19 infection and even pst COVID-19 vaccination must prompt proper investigation, and other causes of neurologic symptoms must first be ruled out prior to considering the vaccine as the primary cause [15]. 

The present study was limited to only patients newly diagnosed with PIMN, exemplified by GBS and its variants, via electrodiagnosis between 2018 and 2021 in a single center. Our study included only newly diagnosed cases from 2018 to 2021; cases diagnosed prior to and after this period were not included. Another limitation is that while most of the patients included in 2020-2021 either had COVID-19 or had their COVID-19 vaccination done prior to GBS, this study did not include data regarding COVID-19 testing and vaccination. As of writing, there was limited data on how COVID-19 can lead to peripheral neuropathies.

## Conclusions

During the initial two years (2020-2021) of the COVID-19 pandemic, our study showed an increased incidence of GBS as compared to other types of PIMN such as CIDP in the Metropolitan Medical Center. The researchers speculate that COVID-19 may somehow play a role in the increasing incidence. However, further studies correlating the COVID-19 status of the patient with their clinical symptoms are still needed to confirm the study. DM neuropathy on the other hand still is the most common cause of peripheral neuropathy. It was mostly seen among male patients above 60 years of age. However, in this study, DM neuropathy showed a significant decrease in incidence during the COVID-19 pandemic. This may be due to a decrease in the number of consultations with a physician during the pandemic.

A multicenter study is also recommended to have a more validated result of the incidence. Future research can also include COVID-19 testing, vaccination status, and electrophysiological testing for patients diagnosed with COVID-19 presenting with neurologic abnormalities. While this study only focused on identifying the incidence of GBS and other peripheral neuropathies seen in Metropolitan Medical Center, the researchers aimed to increase awareness of the possible neurologic sequelae of the COVID-19 virus. it is important to take note that not all COVID-19-infected individuals present with pulmonary symptoms but also present with neurologic abnormalities such as GBS.
